# Advancing state estimation for lithium-ion batteries with hysteresis through systematic extended Kalman filter tuning

**DOI:** 10.1038/s41598-024-61596-0

**Published:** 2024-05-30

**Authors:** J. Knox, M. Blyth, A. Hales

**Affiliations:** 1 Faculty of Engineering, University of Bristol, Bristol, BS8 1TR UK; 2 The Faraday Institution, Quad One, Becquerel Avenue, Harwell Campus, Didcot, OX11 0RA UK

**Keywords:** Electrical and electronic engineering, Batteries

## Abstract

Knowledge of remaining battery charge is fundamental to electric vehicle deployment. Accurate measurements of state-of-charge (SOC) cannot be obtained directly and estimation methods must be used instead. This requires both a good model of a battery and a well-designed state estimator. Here, hysteretic reduced-order battery models and adaptive extended Kalman filter estimators are shown to be highly effective, accurate predictors of SOC. A battery model parameterisation framework is proposed, which enhances standardised methods to capture hysteresis effects. The hysteretic model is parameterised for three independent NMC811 lithium-ion cells and is shown to reduce voltage RMS error by 50% across 18 h automotive drive-cycles. Parameterised models are used alongside an extended Kalman filter, which demonstrates the value of adaptive filter parameterisation schemes. When used alongside an extended Kalman filter, adaptive covariance matrices yield highly accurate SOC estimates, reducing SOC estimation error by 85%, compared to the industry standard battery model.

## Introduction

Lithium-ion batteries are currently the preferred power technology for hybrid and electric vehicles (EV) due to their high energy density, efficiency, and extended lifespan^1^. Advanced battery management systems (BMS) are required to enhance the safety and longevity of lithium-ion cells, and apply corrective measures when operational limits are exceeded. Accurate real-time estimation of state-of-charge (SOC) is a critical task for the BMS. At present, EVs are the dominant application for BMSs. These systems require robust and accurate SOC estimators to maximise battery lifetime performance and range, and to meet consumer demand for reliable driving range predictions^2^.

Direct SOC measurement techniques, such as Coulomb counting, are adversely affected by poor initialisation, drifts resulting from current sensor noise, and variations in cell capacity due to changes in state-of-health and temperature^3^. Open-circuit voltage (OCV) may be used to infer SOC via lookup tables^4^. However, long relaxation periods are required to approximate OCV, rendering it impractical for online state estimation. The presence of hysteresis also significantly reduces the accuracy of this approach^5^. Data-driven methods such as artificial neural networks^6^ model cell behaviour from measured data without prior knowledge of cell properties; these machine learning algorithms typically suffer from poor generalisation and thus require extensive datasets to achieve sufficient accuracy.

Cell SOC may be implicitly derived from accessible measurements such as terminal voltage, current, and surface temperature, using mathematical models. In principle, a selection of models may be deployed for this purpose^7^. Electrochemical models consider fundamental interactions at the particle scale within the electrodes^8^. Although capturing the underlying physics, these models require more computational resources than are typically available in a BMS, and extensive parameterisation is necessary to operate the models effectively. Empirical models describe the evolution of cell voltage using relationships to measurable parameters, but can suffer from inaccuracies of 5-20% as key dynamics may not be fully captured^3^.

Equivalent circuit models (ECM) simulate the response of a cell using basic electrical components, such as voltage sources, capacitors, and resistors. The most commonly employed ECM in BMSs is the Thévenin model, typically containing two resistor-capacitor parallel pairs in series with a resistor, and a voltage source representing OCV. The architecture of ECMs is comparatively less complex than physics-based models, making them ideally suited for real-time operation and BMS applications. Whilst ECMs do not describe intrinsic physical processes as with electrochemical models, they are significantly easier to parameterise accurately^9,10^. The accuracy of an ECM is heavily dependent on the quality of its parameters, so numerous parameterisation methods have been proposed^11,12^. In all cases, the voltage response of a cell is recorded experimentally under a particular current-based loading profile. The galvanostatic intermittent titration technique (GITT) is typically used to fit ECM voltage responses, with numerical algorithms optimising the ECM component parameters to minimise error between the experimental data and modelled voltage response. Model parameters vary with temperature and SOC, so parameterisation and experiments must explore a suitable a range of temperatures and charges. Voltage hysteresis is known to cause considerable error in ECMs and subsequent SOC estimation^13^, yet the widely adopted Thévenin model architecture is unable to account for its effects. Previous studies include additional circuit components to account for hysteresis^3^, however a standardised time-domain based parameterisation technique for ECMs with hysteresis is not reported in the published literature^14,15^.

In BMS applications, cell voltage informs predictions of SOC through algorithms such as the extended Kalman filter (EKF). Kalman filters combine measured data with a model to directly compute SOC and other relevant cell state variables. The combination of both modelled and measured data produces a more accurate SOC estimate than could be obtained from either individually. The EKF is applied to nonlinear systems and provides an approximate solution to the optimal state-estimation problem^16^.

The parameters of a Kalman filter must be chosen carefully to reflect both the cell of interest and the available data. Filters can be designed such that their parameters are updated automatically to reflect the current operating conditions and model parameters of a cell. This is referred to as an adaptive filtering scheme ^17,18^. Many adaptive estimators have been proposed, varying in choice of numerical methods and underlying models^19–21^. Similarly, filtering methods have been generalised to estimate both SOC and auxiliary measures such as state-of-health and remaining useful life^22–24^.

Despite the extensive literature on adaptive Kalman filtering, appropriate tuning of EKF parameters remains a difficult task. Inadequately tuned filters may produce unstable results and time-consuming empirical methods, although widely adopted in literature, cannot be generalised to other models^25^. Systematic determination of EKF parameters for ECM applications have been proposed to account for current sensor noise and model parameter errors^26^. While hysteresis models and EKFs are used variously throughout the literature, the combination of adaptive filters, systematic tuning, and hysteretic cell models have not been experimentally validated for SOC estimation. Simplicity-accuracy trade-offs remain unexplored in such cases. Here, we address this literature gap by exploring the implementation methods and accuracy gains that such an approach can offer. We build on recent work^26^ to show the potential accuracy gains when hysteresis models and experimental parameterisation methods are combined with systematically tuned adaptive EKFs through careful validation on experimental datasets.

This study explores and introduces a novel time-domain-based approach to ECM parameterisation with hysteresis, in order to investigate the benefits to model accuracy that can be achieved through the inclusion of a hysteresis element within an ECM. Further, the investigation sets out to define a method to systematically tune EKF parameters for hysteretic battery models, where performance and viability of the process are validated through comparison with experimental data. The implementation of dynamic EKF parameters is hypothesised to increase the accuracy of SOC estimation when validated on experimental measurements. The investigation is motivated by the benefit that can be gained by BMS manufacturers to improve the accuracy of SOC estimation and refine tolerances on cell operational limits. This has the potential to increase accessible energy for a battery and enable enhanced battery control algorithms to mitigate operational scenarios which cause rapid degradation. Future research directions are proposed to further enhance hysteretic SOC estimation.

## Methods

The following section details the hysteresis model architecture, model parameterisation methods and adaptive Kalman Filter tuning methodology.

### Modelling

**Figure 1:**
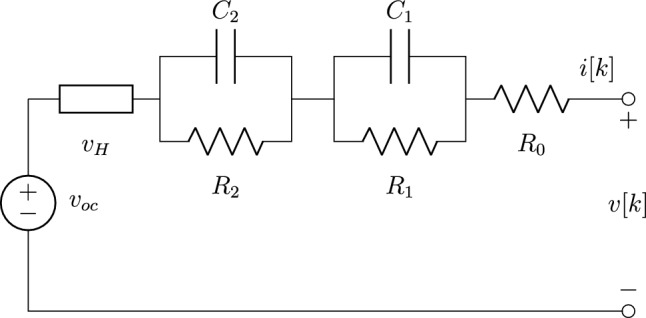
Proposed enhanced self-correcting equivalent circuit model. Includes an ideal voltage source $v_{oc}$, equivalent series resistance $R_0$, two resistor-capacitor (RC) networks and hysteresis voltage element $v_H$^27^.

This study benchmarks expected ECM performance using a standard Thévenin model with two resistor-capacitor parallel pairs in series with a resistor. The proposed enhancement to the standard Thévenin model, tested for suitability in this study, is the enhanced self-correcting model (ESC) introduced by Plett^27^. This connects the conventional Thévenin model in series with a single-state hysteresis voltage component $v_H$. The ESC model architecture is presented in Fig. 1. Here, $R_0$ represents the equivalent series resistance and each resistor-capacitor (RC)-network captures the effects of diffusive electrochemical behaviours, with time constants $\tau _j = R_jC_j$. The quantity $v_{oc}$ represents cell OCV, critically, without capability to consider any hysterical component of OCV. As outlined below; this effectively summarises the limitations of the standard Thévenin architecture in the context of considering hysteresis. The hysteresis voltage element is defined by $v_H = Mh$, where *M* is maximum hysteresis voltage, and *h* is a unitless hysteresis state bounded between $-1$ and 1. The hysteresis voltage element ($v_H$) represents the difference between the ESC and a standard Thévenin model (which includes just the two resistor-capacitor parallel pairs in series with a resistor). This component allows for the inclusion of more complex variability in over-potential (during kinetic loading) and deviation from the absolute value of the voltage source ($v_{oc}$) during open circuit periods. Here, OCV is modelled as a non-constant value at a given SOC and temperature, calculated by combining the magnitude of $v_H$ with the magnitude of $v_{oc}$.

A discrete-time representation of the ESC is necessary for deployment in lightweight BMS controllers^15^. The discretisation scheme requires model inputs to be uniformly sampled in time, and current *i* to be constant over the sampling period $\Delta t$. The latter necessitates sampling duration $\Delta t$ to be small relative to the timescale of battery operation. The discrete-time ESC state equation is a map describing the evolution of model variables over time index *k*, including SOC $z_k$, RC-network currents $i_{R_j,k}$, and the hysteresis state $h_k$. Dynamics are given by

1 $$\begin{aligned} z_{k+1}&= z_k -\frac{\eta _k \Delta t}{Q} i_k, \end{aligned}$$

2 $$\begin{aligned} i_{R_j,k+1}&= \alpha _{RC_j} i_{R_j,k} + (1-\alpha _{RC_j}) i_k, \end{aligned}$$

3 $$\begin{aligned} h_{k+1}&= \alpha _{H,k} h_k + (A_{H,k} - 1) \textrm{sgn}(i_k), \end{aligned}$$

where *j* is the index of the RC-network, $\eta$ is cell efficiency, *Q* is cell capacity, $\alpha _{RC_j} = \textrm{exp} (-\Delta t / \tau _j)$ is the RC-subcircuit rate factor and $\alpha _{H_k} = \textrm{exp} (-| i_k \eta \gamma \Delta t / Q |)$ is the hysteresis evolution factor with rate constant $\gamma$. The ESC output equation gives the terminal voltage of the cell $v_k$ at time *k* as

4 $$\begin{aligned} v_k = v_{oc}(z_k,T_k) + Mh_k - \sum _j R_j i_{R_j,k} - R_0 i_k~, \end{aligned}$$

where *T* is temperature. This work assumes $\eta =1$.

### Experimental setup

The principle focus of this investigation was to evaluate the performance benefits of the hysterical model with our novel parameter estimation methods. The performance of the models developed and parameterised in this work is entirely dependent on the experimental data generated. To this end, we now introduce the experimental setup and provide a complete description of the parameterisation experiments that were conducted.

**Figure 2:**
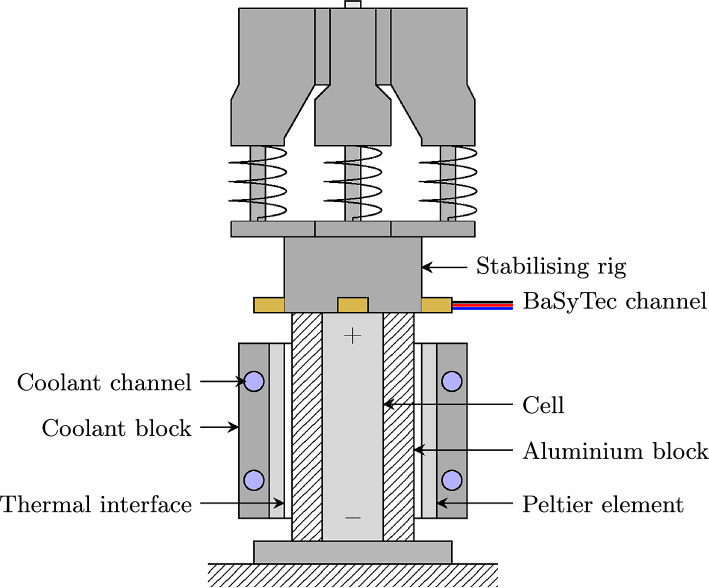
Experimental setup for a single cell connected to a channel on the *BaSyTec CTS* battery cycler (not shown) and thermally controlled using two side-mounted Peltier elements and a thermal controller (not shown).

All experiments were conducted on 5 Ah *LG Chem M50LT* 21700 cylindrical lithium-ion cells, which comprise an NMC cathode and graphite-silicon anode^28,29^ and have previously been used in relevant publications related to EV BMSs^30,31^. Three cells were used for the tests, with each cell subjected to all procedures, resulting in three complete datasets. The cells were at beginning of life at the start of the experiments. Coupled with the uniform temperature of 25 °C (see below) used across all tests (mid-range temperature across the cell’s temperature operating window), this ensured each cell behaved in a similar manner and responded in the same way to a given kinetic excitation. This was important for data analysis in order to demonstrate the robustness of the processes introduced by the present study. All experiments were conducted using a 4-wire connection *BaSyTec CTS* battery cycler, supporting 1 mV voltage precision, 1 mA current precision^32^ and a data sampling rate of 10 Hz, which was used throughout. The procedures were conducted at a cell surface temperature of 25 °C maintained to within $\pm \;$0.25 °C using cell-surface mounted Peltier elements driven by thermal control hardware. A schematic of the experimental setup is shown in Fig. 2. Coaxial probes were used to make an electrical connection to the positive terminal and a bespoke annular connector was used to make the negative connection to the ‘shoulder’ of the cell, mimicking battery pack assembly processes used in the EV industry. The stabilisation rig maintained uniform and repeatable pressure applied from the electrical connections onto the cell, ensuring traceability across each test setup and each cell.

### Experimental procedures

Coulombic counting is relied upon to extract the real-time SOC from the experimental data. It was therefore important that all experiments were conducted in isolation so that any uncertainty in the initial SOC was not able to accumulate over subsequent tests. As such, a standardised ‘SOC reset’ procedure was employed between each test, and always ran at 25 °C.

SOC reset procedure:

1. 0.3 C constant current charge to 4.2 V (the upper voltage limit of the cell);
2. Constant voltage hold at 4.2 V, until $i$ < 0.01 C;
3. Rest for 4 h to reach electrochemical equilibrium.

Four experiments were required to parameterise and test the ESC model. The following procedures were conducted on each of the three cells under test:

- GITT;
- GITT for OCV;
- GITT with charge pulse;
- WLTP cycle.

**Figure 3:**
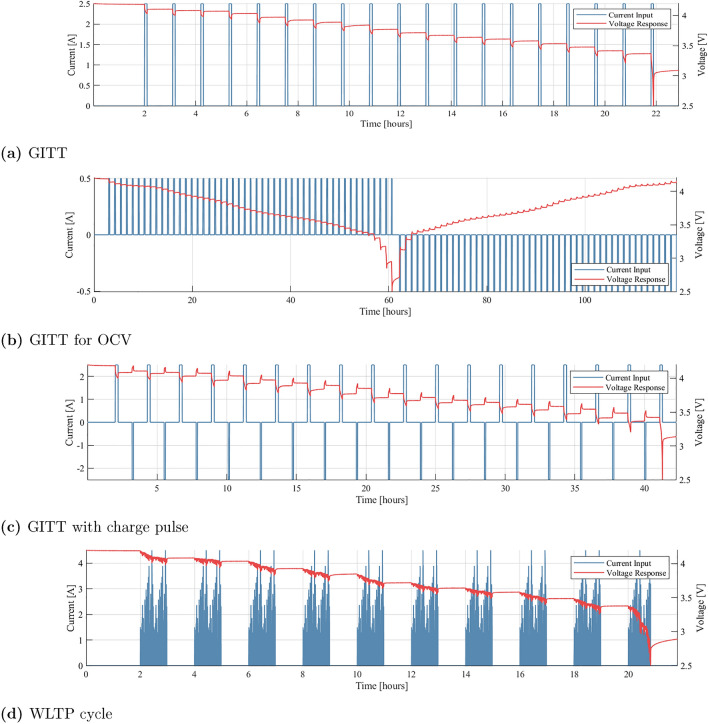
Example experimental data taken directly from each of the four tests completed on each cell. Data from Cell A is shown here. Current (*I*) > 0 A signifies a discharge of the cell.

These procedures are now introduced, with examples from Cell A plotted in Fig. 3.

**GITT.** A series of constant current discharge pulses and relaxation periods. In this study, 6 minute 0.5 C constant current discharge pulses were used to reduce SOC by 5 % during each titration, with 1 h rests between each discharge pulse. The procedure was initiated with the cell at 100 % SOC and repeated until the the lower voltage limit of the cell (2.5 V) was reached.

**GITT for OCV.** Following Birkl *et al.*^33^, 12 minute, 0.1 C constant current discharge pulses were used to reduce SOC by 2 % during each titration, with 1 h rests between each discharge pulse. The procedure was initiated with the cell at 100 % SOC and repeated until the the lower voltage limit of the cell (2.5 V) was reached. The procedure was then reversed, utilising 0.1 C charge pulses, repeated until 4.2 V was reached.

**GITT with charge pulse.** An alternating discharge-charge GITT procedure. Discharge consisted of a 12 minute 0.5 C constant current discharge pulse to reduce SOC by 10 %, followed by a 1 h rest. The cell was then charged for 6 minutes at 0.5 C, increasing SOC by 5 %. The procedure was initiated with the cell at 100 % SOC and repeated until the lower voltage limit (2.5 V) was reached.

**WLTP cycle.** For model validation and performance quantification, a drive cycle incorporating the Worldwide Harmonised Light Vehicle Test Procedure (WLTP) was used^34^. To determine the power required for an individual cell from the WLTP, the Driving Cycle block was used within Mathworks’ Simulink software^35^, alongside simple vehicle dynamics representing an arbitrary EV. Following the process set out by Hales *et al.*^36^, 10% of the battery was discharged over a 1 h current-based loading profile, followed by a 1 h rest. This procedure was then repeated until the lower voltage limit (2.5 V) was reached. This is designed to represent an EV completing a number of 1 h journeys with short rests in between, allowing the applied validation protocol to evaluate both kinetic and rest-period SOC estimation. Whilst a current-based drive-cycle was desirable for the experimental work, the resulting experimental power profile was used for model validation. This represents real-world conditions whereby Coulomb counting cannot be readily applied.

### Parameter identification

Cell OCVs were identified from the GITT for OCV experiments. To account for hysteresis, measurements were averaged over the discharge and charge components of the procedure.

For given RC time-constants, parameters $R_0$, $R_1$, $R_2$, $C_1$ and $C_2$ of the standard Thévenin model (without hysteresis) may be identified from GITT data with ordinary least-squares, using established methodologies from the literature^30,37–39^. Hysteresis parameters cannot be identified in the same way, and must instead be determined through nonlinear optimisation. Parameter values of any given ECM component will change when hysteresis is included due to the overpotential contribution of the hysteresis element. Thus, ESC model parameters cannot be transferred over from a linear least squares parametersiation of an ECM. Instead, all parameters must be identified through nonlinear optimisation. As the overpotential contribution of the hysteresis element is small ESC (with hysteresis) component parameters may be well-approximated by their ECM (without hysteresis) equivalents. The ECM parameters were therefore be used as an initial solution for the nonlinear parameterisation. When combined with local gradient-based numerical optimisers, this allows ESC parameters to be identified quickly and accurately.

Initially, Thévenin ECM parameters were fitted to GITT data using ordinary least squares. Time constants $\tau _1=2$ and $\tau _2=50$ were chosen based on previous works^12,40^. Parameters of each GITT pulse were used to populate a lookup table, with linear interpolation used between breakpoints. The resulting parameters were also used as initial conditions for a nonlinear optimisation procedure. A numerical optimisation routine was used to identify parameters $R_0$, $R_1$, $R_2$, $\tau _1 = R_1 C_1$ and $\tau _2 = R_2 C_2$ at each SOC break-point in the lookup table for a given value of $\gamma$. The resulting least-squares error quantifies the best-possible ECM model-fit for that value of $\gamma$. A further numerical optimisation routine was used to identify the optimal $\gamma$ that minimises this error. To capture changes in the hysteresis state, GITT with charge-pulse data were used.

To optimise, hysteresis overpotential $M$ was first computed from GITT for OCV data as

$$\begin{aligned} M(\textrm{SOC}) = 0.5\left[ v_{oc}^{\textrm{charge}}(\textrm{SOC}) - v_{oc}^{\textrm{discharge}}(\textrm{SOC})\right] . \end{aligned}$$

Once $M$ was computed, nonlinear parameter optimisation was performed using MATLAB/Simulink Design Optimisation^41^. Simscape electrical components were used to model standard circuit elements. At each timestep, a Coulomb counter updated SOC and ECM parameter values were extracted from the latest optimiser solution-guess. An output voltage was then computed from this and the associated residual passed to a numerical optimisation algorithm to optimise component values at any given SOC. Gradient-based nonlinear least-squares optimisers are preferable due to fast convergence rates^42,43^. Separate estimation tasks were performed at each lookup table breakpoint.

Parameter bounds were required to ensure a well-posed identification problem^44^. To isolate the effects of each RC-network, time constants must be constrained to disjoint ranges. The chosen parameter constraints were $0.5 \; s \le \tau _1 \le 25 \; s$, $50 \; s \le \tau _2 \le 500 \; s$. Sensible parameter value ranges can be estimated from previous studies^45^, with the chosen constraints selected through trial-and-error based on the observed transient decay rates in GITT data. The specific values of each parameter constraint are not critical to the final results, as constraints are only used to place an ordering on RC pairs, so that $\tau _1 < \tau _2$; this ordering is necessary to ensure the identifiability of parameters^44^. Notably, it was found that if $\tau _1 < 0.5$ s, the optimiser would associate the instantaneously acting ‘series’ resistance with $R_1$ as well as $R_0$, leading to unstable parameters with large variance from one SOC bound to the next.

### Analysis of the hysteretic parameterisation method

Summarising the above, the presented parameterisation method proceeds by:

1. Fitting model parameters using ordinary least squares, with fixed RC time-constants and without hysteresis;
2. Updating model parameters and RC time-constants using a nonlinear optimiser, with the inclusion of a hysteresis term with fixed rate-constant $\gamma$;
3. Numerically optimising over both the model parameters and $\gamma$, to parameterise the full model.

This three-step process incurs distinct computational advantages when compared to a single-step identification procedure. By leveraging prior estimates of RC time-constants, the least-squares parameterisation of step (1) can propose sensible model parameters near instantaneously. Accuracy is increased in step (2) by no longer fixing the RC time constants. As the optimal solution of step (2) is necessarily similar to that of step (1), the performance and robustness of the gradient-based solver is greatly improved by initialising with the least-squares parameters. Similarly, the parameters found in step (2) — numerical optimisation with constant hysteresis-rate — will be close to the optimal parameters of the full ESC model. Thus, by gradually re-optimising parameters over increasing model complexities, an optimal solution is found for the parameter identification problem, with significant increases in robustness and computational efficiency when compared to solving for full model parameters directly, in a single step.

### Kalman filtering for SOC estimation

Kalman filters have been covered extensively in the literature^25,46–48^, including detailed discussions for lithium-ion battery SOC estimation^26,49–51^. We follow the EKF implementation proposed by Rzepka *et al*.^26^; a short summary is provided here.

A state quantifies the instantaneous configuration of a dynamical system. As per Eqs. (1)–(3), the state vector of the ESC at time $k$ is $x_k = [z_k, i_{R_j, k}, h_k]$. Given an initial state $x_0$, a model, and current draw $i(t)$, the state of the lithium-ion cell at some future time may be computed. However, computed states may diverge from actual system states due to modelling or sensor errors. Combining modelled state estimates ${\hat{x}}_k^-$ with measured data $y_k^-$ provides a means to correct this error. Kalman filters are an optimal state estimation algorithm for achieving this goal. Filtered state estimates ${\hat{x}}_k^+$ provide explicit SOC inferences and do not require the BMS to perform any post-processing to extract SOC from the results. Kalman filters require linear system dynamics and therefore cannot be applied to the ESC, as the hysteresis term is nonlinear in current-draw $i$. The extended Kalman filter generalises the method to nonlinear systems by taking a linear approximation of the system dynamics at each step. While this does not guarantee optimality, it often works well in practise when filters are properly tuned^25^.

Uncertainties in modelled and measured data are quantified through covariance matrices $\Sigma _{{\hat{x}},k}^-$ and $\Sigma _v$, respectively. Unfiltered data ${\hat{x}}_k^-$ and ${\hat{y}}_k^-$ are combined and weighted according to their respective uncertainties to produce a state estimate ${\hat{x}}_k^+$ that minimises the $\ell _2$ state estimation error. In addition, the covariance matrix $\Sigma _{{\hat{x}},k}^-$ is also updated in light of available data, resulting in a covariance matrix $\Sigma _{{\tilde{x}},k}^+$ that captures uncertainty in ${\hat{x}}_k^+$. As noted, SOC is available directly from the state estimate ${\hat{x}}_k^+$.

Tuning a Kalman filter involves selecting appropriate values for the initial state estimate ${\hat{x}}_0^+$, state covariance $\Sigma _{{\tilde{x}},0}^+$, measurement noise covariance $\Sigma _v$, and process noise covariance $\Sigma _w$. Accurate uncertainty tuning is critical for successful implementation of an extended Kalman filter^25^. Recent work has demonstrated how filters can be tuned systematically for SOC estimation in hysteretic battery models^26^. Two types of filter are proposed. An adaptive filter selects measurement and process covariance matrices according to the instantaneous SOC and current-draw, in much the same fashion as an ECM chooses component parameter values for the current SOC; a non-adaptive filter uses the same measurement and process covariance matrices across the entire depth of discharge. We consider both non-adaptive and adaptive approaches here, implemented using the methodology presented by Rzepka* et al*.^26^.

## Results and discussion

In this section, several variants of the standard Thévenin ECM, parameter identification, and SOC estimation processes are introduced, as summarised below:

- **ECM:** standard Thévenin ECM, with ordinary least-squares parameterisation;
- **ECM-opt:** ECM with nonlinear optimisation for parameter identification;
- **ECMh:** ESC (Thévenin ECM with a hysteresis component) and ordinary least-squares parameterisation;
- **ECMh-opt:** ECMh with nonlinear parameter optimisation;
- **Constant-EKF:** extended Kalman filter SOC estimator, with $\Sigma _v$ and $\Sigma _w$ constant across depth of discharge;
- **Adaptive-EKF:** EKF with $\Sigma _v$ and $\Sigma _w$ varying according to estimated SOC, and current draw $i(t)$.

**Figure 4:**
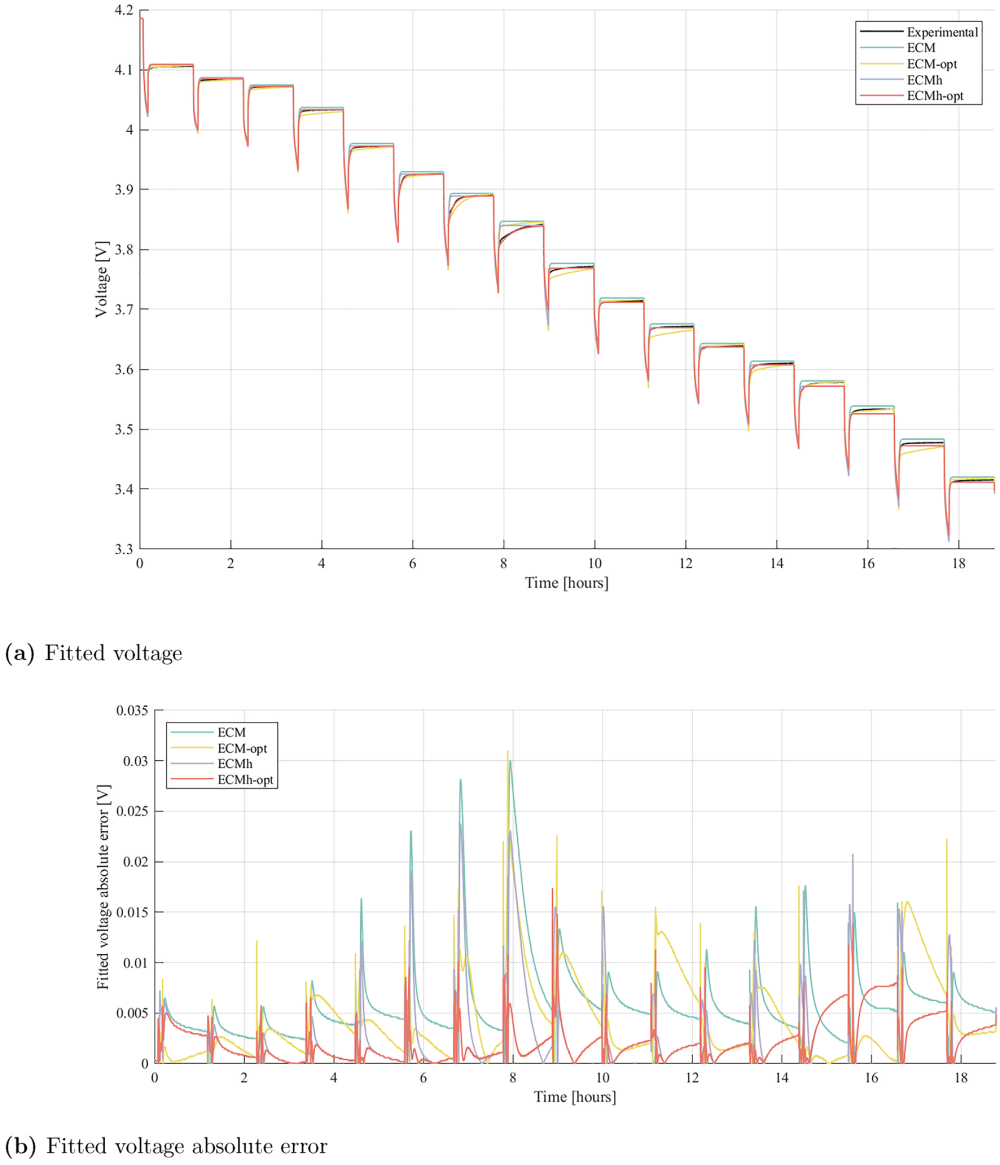
Experimentally measured voltage from the GITT experiment on Cell A, compared to the fitted voltage for the ECM, with and without parameter identification optimisation and with and without hysteresis.

Figure 4 illustrates the results of the fitting procedures outlined in the methodology section. Model fits are shown on GITT data from cell A, which was used to parameterise a heirarchy of increasingly accurate models. Firstly, a Thévenin model was parameterised from the data, labelled ‘ECM’, using ordinary least-squares with $\tau _1=2$ s and $\tau _2=50$ s. Next, the ECM parameters were refined using the numerical optimisation routine discussed in the methodology section. Circuit component paramters, including timescales $\tau _1$ and $\tau _2$, were fitted to the observed data to give a better model fit, labelled ‘ECM-opt’. Separate charge and discharge OCV curves were computed from GITT-for-OCV data, which are used in conjunction with ordinary least-squares parameterisation and the ESC model, giving a hysteretic ECM labelled ‘ECMh’. Finally, GITT and GITT-with-charge-pulse data were used to optimise the ECMh parameter values, labelled ‘ECMh-opt’. Each step results in a model whose structure or parameters capture more battery dynamics than the previous model, resulting in increasing accuracy. For Cell A, the RMS voltage-fitting error is 7.0 mV for ECM, 6.0 mV for ECM-opt, 4.9 mV for ECMh and 3.0 mV for ECMh-opt.

**Figure 5:**
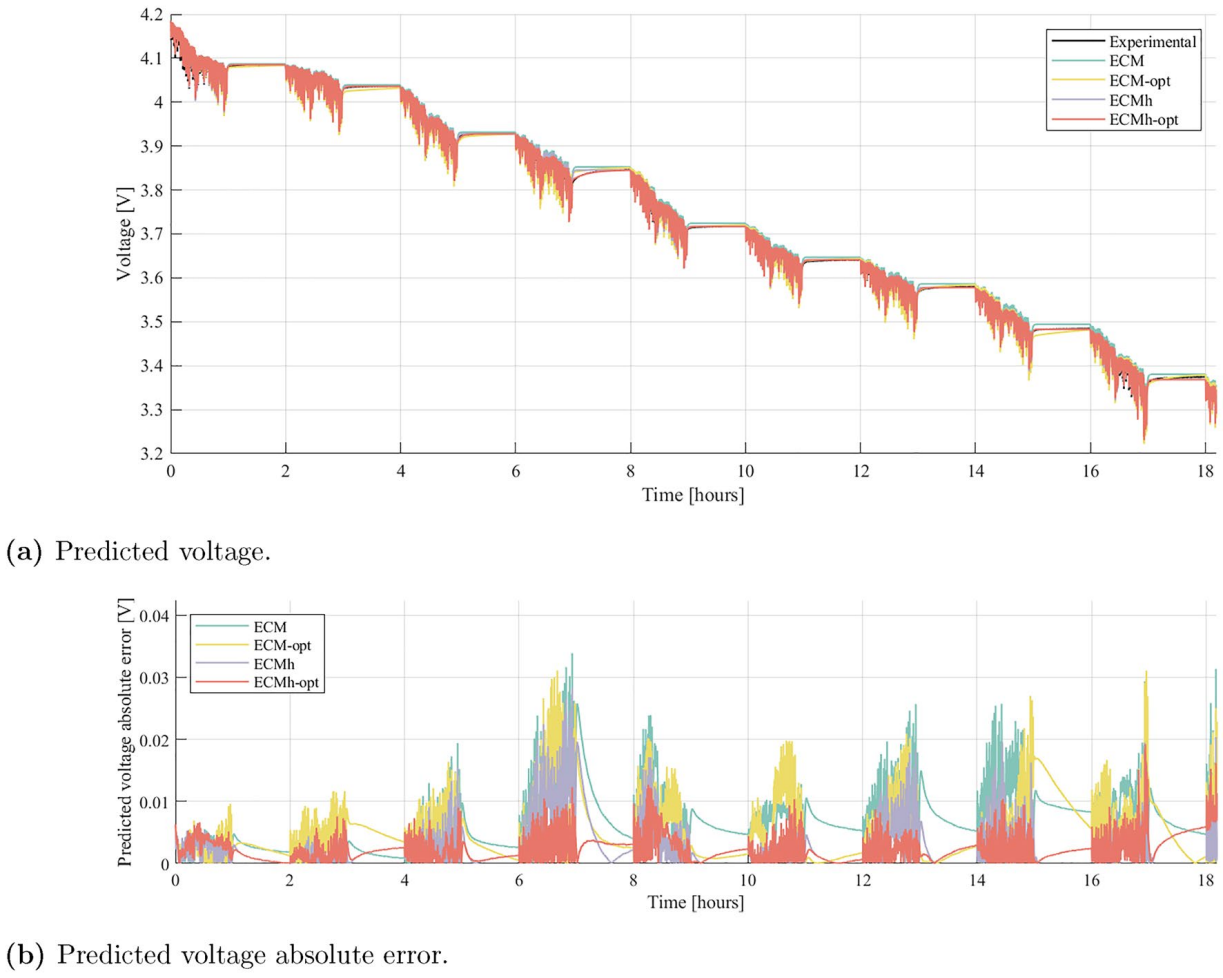
Experimentally measured voltage from the WLTP experiment on Cell A, compared to the fitted voltage for the ECM, with and without parameter identification optimisation and with and without hysteresis.

**Figure 6:**
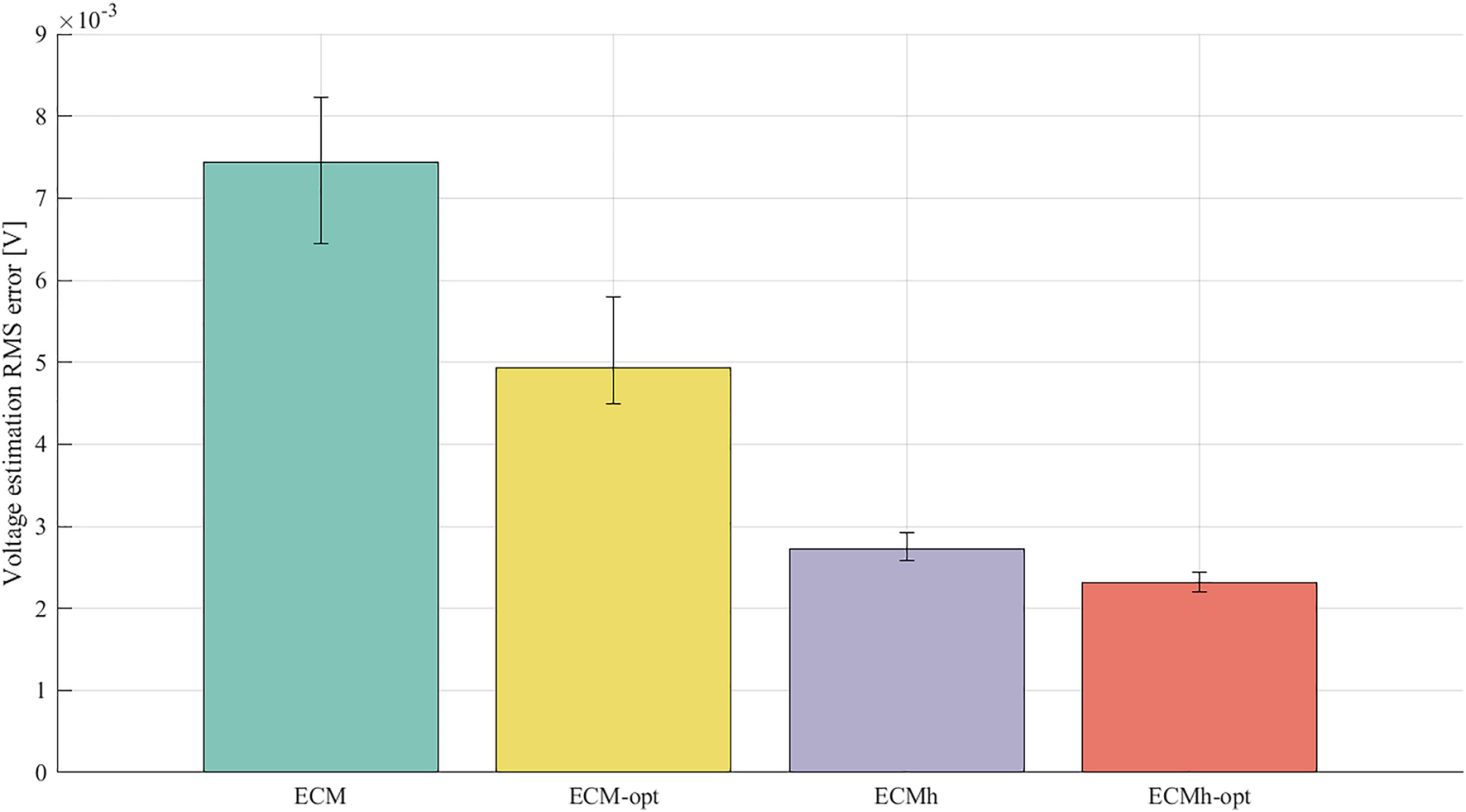
The mean RMS voltage prediction error for all models of each cell tested, evaluated against the WLTP drive cycle. Error bars report the maximum and minimum RMS voltage prediction error for each of the models.

Figure 4 provides evidence that the optimised parameter identification process and the hysteresis component result in better fits to the parameterisation data. Nevertheless, comprehensive validation requires the model performances to be evaluated against an independent dataset. Validation results are shown in Fig. 5, where each model is evaluated on the developed WLTP drive cycle. Time series of voltage predictions and absolute errors are plotted. In addition, the RMS voltage errors from the WLTP validation process are displayed in Fig. 6, which shows the average RMS errors over all cells, with maximum and minimum cell RMS errors displayed as error bars.

Benefits are clearly obtained from the optimisation-based parameterisation and the inclusion of a hysteresis component. Significant reductions in absolute error are observed in both the dynamic periods, where load is on the cell, and during periods of rest. Across all cells, a common trend is present — optimising parameter identification processes or including a hysteresis component within the ECM enhances the performance of the model. Furthermore, error bars are seen to shrink as model accuracy improves. This indicates that while the performance of simple ECMs can fluctuate substantially from cell to cell, hysteresis elements and better parameterisation creates more robust models.

It is noted that a small steady state error remains; when using ECMh-opt, the predicted return to OCV carries more error at some SOCs than ECM-opt. This was consistent across all cells, suggesting that small errors exist in the parameterised OCV curves. Such errors can arise from the GITT for OCV procedure, where rest times are too short for the cell to reach equilibrium. This suggests that yet higher accuracy may be achievable if rest-times are further increased during parameterisation experiments. Lookup tables for OCV could also be included in the optimisation procedure, so that the numerical optimiser can correct errors in experimental OCV measurements. Results also suggest that the parameter optimisation process in ECM-opt can help to eliminate rest-OCV error. Nevertheless, parameters that are optimised to eliminate rest-OCV error are less accurate at describing kinetic periods, evidenced from the data shown in Fig. 5b. This results in the increased magnitude of the ECM-opt error bar in Fig. 6. To overcome this, separate resting and dynamic parameter tuning could be investigated, using decoupled optimisations for kinetic and rest periods. While this is an interesting avenue for further cell model improvements, we consider it to be beyond the scope of the present study, since it would introduce a third parameter, current $i(t)$ into the lookup table.

### SOC estimation and adaptive extended Kalman filter

Extended Kalman filter (EKF) SOC estimation is validated on the developed WLTP drive cycle data. The following discussion is set against the performance criteria most relevant to the BMS industry — SOC estimation. This allows focus to be set directly on the performance of the EKF tuning methods applied to the various models. For completeness, the exact models discussed in the previous section are used to establish a baseline estimation accuracy, without any EKF inclusion. As the WLTP drive cycle is power-based, this is achieved by using a given model to compute the necessary current draw satisfying the power requirements at any given time-step, and Coulomb-counting on the modelled current-draw to estimate SOC. The method is highly computationally efficient but has no mechanism to access feedback from true measurements made on the cell, meaning it is prone to SOC-drift over a period of time. The EKF tuning methods are tested on the drive cycle using a similar approach, whereby the chosen model is used to calculate the necessary current-draw at each time step in the drive cycle. However, modelled current-draw is then input to an EKF (with specified tuning) for SOC estimation, rather than Coulomb counting as used for baseline estimation accuracy.

**Figure 7:**
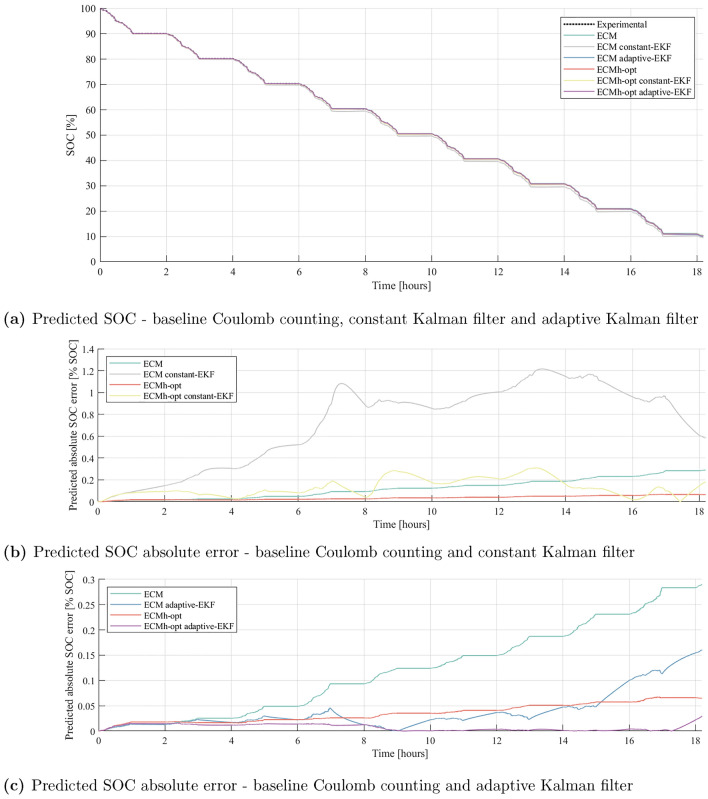
Experimentally measured SOC from the WLTP experiment on Cell A, compared to the predicted SOC from ECM and ECMh-opt, through Coulomb counting, constant and adaptive Kalman filtering.

**Figure 8:**
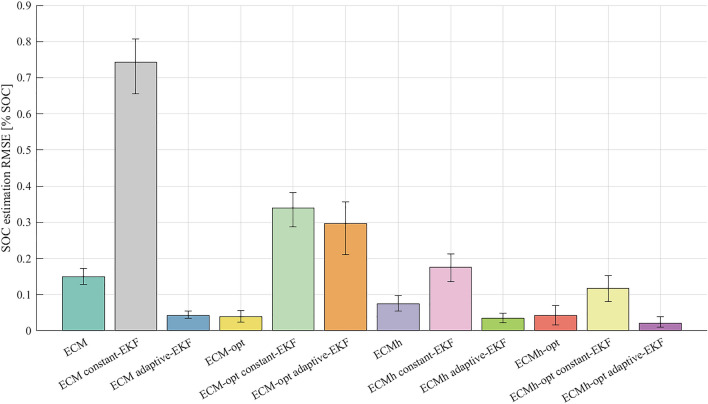
The mean SOC prediction error for all models of each cell tested, evaluated against the WLTP drive cycle. Error bars report the maximum and minimum RMS SOC prediction error for each of the models.

Each model is tested with both constant and adaptive EKF tuning, and compared against true cell SOC calculated via Coulomb counting. Results are depicted in Fig. 7, showing time-series results for cell A with the ECM and ECMh-opt models, and in Fig. 8, depicting RMS error in SOC estimation for every combination of model and EKF tuning. Figure 8 shows the mean RMS error in SOC estimation computed across all cells, with error bars indicating the cells with the smallest and largest estimation errors.

Observing Fig. 8, the Coulomb counting method is shown to outperform the constant-EKF method for all introduced model architectures and parameter tuning methods. This highlights a clear limitation in the constant parameter EKF method - the additional computation required by this method does not offer a reduction in SOC estimation performance. Focusing on the specific case of Cell A, the poor performance of constant-EKF is clear in Fig. 7b. However, the Coulomb counting method is also seen to be limited in Fig. 7c. The magnitude of SOC estimation error can be seen to increase as the drivecycle progresses (for both ECM and ECMh-opt), to the extent that an SOC estimation error of 0.29 % exists by the end of the 18 h cycle. This is likely caused by the lack of feedback mechanism when Coulomb counting through the model and it is reasonable to expect that this error will continue to grow over longer drive cycles and repeated charge-discharge cycles. This problem could be alleviated by integrating a feedback mechanism into the model. For example, by cross-referencing against a SOC-OCV lookup table at regular intervals. However, this would increase the complexity of the SOC estimation method and reduce the attraction of the lightweight modelling method.

The adaptive-EKF tuning is shown to perform very effectively for the ECM, ECMh and ECMh-opt models, reducing SOC estimation error in all cases (Fig. 8 and, crucially, mitigating the issue of drift that limits the Coulomb counting method, evidenced in Fig. 7c). Firstly, this emphasises the importance of high quality, adaptive-EKF tuning for effective SOC estimation in both standard ECMs and those including hysteresis components. Secondly, this highlights that for the NMC811 cells used in this study, the development of effective adaptive-EKF tuning methods should be considered a higher priority than the inclusion of hysteresis components and development of hysteresis parameterisation methods. With the most basic ECM, the adaptive-EKF tuning method is able to significantly reduce SOC estimation error, below what is achieved with the inclusion of hysteresis terms and parameter optimisation methods. For other cell chemistries, particularly LFP cells which are known to have significant hysterical components within their OCV, this statement must be validated before the development of SOC estimation methods for a BMS.

The simplest model tested here is the ECM without hysteresis, parameterised using ordinary least-squares. Counterintuitively, the combination of this ECM with an adaptive EKF outperforms the optimised ECM for SOC estimation. As time constants $\tau _j$ are allowed to change during the optimisation procedure, there is more variation between the parameters of each ECM-opt model than there is in the parameters of the ECM model. Higher parameter variances causes the EKF to assume greater process noise. As a result, the EKF has greater uncertainty in the ECM-opt SOC estimate and, despite its better parameter set, places less weight on the model results. Thus, greater fluctuation in SOC estimation error is expected, due to the EKF underweighting model predictions whilst filtering and relying more on noisy measurements. This further suggests that accurate characterisation of parameter variances is critical to adaptive EKF accuracy. Given that sample variance is a random variable, a more accurate estimate of parameter variances would be obtained if more than three cells were tested. Ideally, a larger number of cells would be parameterised, resulting in a more meaningful estimate of the parameter variances and process noise covariance matrix. Whilst the performance of the adaptive-EKF SOC estimation methods is very strong in this study, a greater number of cells would certainly make the process more robust, with reduced chance of parameter error caused by singular erroneous results.

## Conclusions

Parameterisation of lithium-ion cell equivalent circuit models is well-established. Here, these methods are generalised to parameterise models with hysteresis, with the introduction of short charge steps to decouple hysteresis from other electrochemical phenomena observed during the completion of a kinetic parameterisation procedure (GITT). Results demonstrate that a well-parameterised hysteresis model reduces RMS voltage prediction error by approximately 50 % on relevant test data, when compared to ordinary least-squares parameterisation of models without hysteresis, as commonly employed in the literature.

Accurate cell models are desirable for control purposes in BMSs, with SOC estimation as a key motivation. Adaptive extended Kalman filtering has been proposed for estimating SOC in models with hysteresis^26^, however the method has not previously been validated experimentally. This important validation is provided here, which is used to demonstrate that a well-parameterised hysteresis model provides very high SOC estimation accuracy when paired with an adaptive extended Kalman filter.

Results show that better cell models will typically yield more accurate SOC estimates. Furthermore, improvements in the Kalman filter, through inclusion of adaptive covariances, always leads to improvements in SOC estimation. Improvements to the Kalman filter are seen here to produce more substantial increases in SOC estimation accuracy, compared to improvements that can be gained by enhancing the underlying model with hysteresis components and parameter optimisation — adaptive covariance matrices reduce SOC estimation error by up to 85 %. It is important that this statement is tested on other cell chemistries, particularly those that are known to have a considerable hysterical component within their OCV, such as LFP cells.

Future work should focus on refinements to extended Kalman filter estimation. A key step towards this is accurate estimation of parameter variances when generating the process noise covariance matrix of an adaptive extended Kalman filter. A further avenue to explore is the inclusion of an explicit temperature dependency in the cell models and associated Kalman filters. Precise thermal control is unachievable in EV applications; future work should therefore extend adaptive Kalman filters to include the effects of cell temperature and heat generation in the process noise covariance matrix. Whilst the impact of temperature on the processes introduced were beyond the scope of the presented study, the processes are expected to be equally effective across the operating range of the lithium-ion cell under test. The same is true for degradation - the processes introduced should be affected for a cell at any state-of-health. As a typical EV battery pack contains many individual cells, efforts should also be made to scale adaptive-EKF methods up to pack-level SOC estimation.

## Data Availability

The datasets used and/or analysed during this study are available from the corresponding authors upon reasonable request.

## Code availability

The code that support the findings of this study are available from the corresponding author upon reasonable request.

## References

[1] Saw LH (2016). Integration issues of lithium-ion battery into electric vehicles battery pack. J. Clean. Prod..

[2] Hannan MA (2017). A review of lithium-ion battery state of charge estimation and management system in electric vehicle applications: Challenges and recommendations. Renew. Sustain. Energy Rev..

[3] Nejad S (2016). A systematic review of lumped-parameter equivalent circuit models for real-time estimation of lithium-ion battery states. J. Power Sources.

[4] Zhang, R. *et al*. A Study on the open circuit voltage and state of charge characterization of high capacity lithium-ion battery under different temperature. *Energies.* **11**(9) (2018). issn: 1996-1073.

[5] Zhang R (2018). State of the art of lithium-ion battery SOC estimation for electrical vehicles. Energies.

[6] Chen J (2018). Neural network-based state of charge observer design for lithium-ion batteries. IEEE Trans. Control Syst. Technol..

[7] Hu, X. *et al.* State estimation for advanced battery management: Key challenges and future trends. *Renew. Sustain. Energy Rev.* **114**(Sept. 2019).

[8] Xiaosong H (2018). Condition monitoring in advanced battery management systems: Moving horizon estimation using a reduced electrochemical model. IEEE/ASME Trans. Mechatron..

[9] Koirala, N. *et al.* Comparison of two battery equivalent circuit models for state of charge estimation in electric vehicles. English. In *Proceedings of the 2015 10th IEEE Conference on Industrial Electronics and Applications, ICIEA 2015*, 17–22 (2015).

[10] Naseri Farshid (2022). An enhanced equivalent circuit model with real-time parameter identification for battery state-of-charge estimation English. IEEE Trans. Ind. Electron..

[11] Hua X (2021). Finding a better fit for lithium ion batteries: A simple, novel, load dependent, modified equivalent circuit model and parameterization method. J. Power Sources.

[12] Birkl, C. R. *et al.* Model identification and parameter estimation for LiFePO4 batteries(2013).

[13] Barai A (2015). A study of the open circuit voltage characterization technique and hysteresis assessment of lithium-ion cells. J. Power Sources.

[14] Huria T (2014). State of charge estimation of high power lithium iron phosphate cells. J. Power Sources.

[15] Plett, G. L. *Battery management systems. Volume I, Battery modeling*. English. Norwood. Artech House (2015).

[16] Simon D (2006). Optimal State Estimation: Kalman, H∞, and Nonlinear Approaches.

[17] Sun F  (2011). Adaptive unscented Kalman filtering for state of charge estimation of a lithium-ion battery for electric vehicles. Energy.

[18] Zhang S (2022). A comparative study of different adaptive extended/unscented Kalman filters for lithium-ion battery state-of-charge estimation. Energy.

[19] Chen L (2022). Adaptive state-of-charge estimation of lithium-ion batteries based on square-root unscented Kalman filter. Energy.

[20] Li W (2020). Electrochemical model-based state estimation for lithium-ion batteries with adaptive unscented Kalman filter. J. Power Sources.

[21] Zhang S (2020). An improved adaptive unscented Kalman filtering for state of charge online estimation of lithium-ion battery. J. Energy Storage.

[22] Zou Y (2015). Combined state of charge and state of health estimation over lithium-ion battery cell cycle lifespan for electric vehicles. J. Power Sources.

[23] Zheng X (2015). An integrated unscented Kalman filter and relevance vector regression approach for lithium-ion battery remaining useful life and short-term capacity prediction. Reliab. Eng. Syst. Saf..

[24] Wassiliadis N (2018). Revisiting the dual extended Kalman filter for battery state-of-charge and state-of-health estimation: A use-case life cycle analysis. J. Energy Storage.

[25] Schneider R (2013). How to not make the extended Kalman filter fail. Ind. Eng. Chem. Res..

[26] Rzepka B (2021). Implementing an extended Kalman filter for SoC estimation of a Li-Ion battery with hysteresis: A step-by-step guide. Energies.

[27] Plett GL (2004). Extended Kalman filtering for battery management systems of LiPB-based HEV battery packs: Part 2. Modeling and identification. J. Power Sources.

[28] Product specification, Lithium Ion INR21700 M50 18.20Wh. LRB-PS-CY18.2Wh-M50. LGC MBD/MBDC. (Aug. 2016).

[29] O’Regan K (2022). Thermal-electrochemical parameters of a high energy lithium-ion cylindrical battery. Electrochim. Acta.

[30] LeBel F-A (2022). Lithium-ion cell equivalent circuit model identification by galvanostatic intermittent titration technique. J. Energy Storage.

[31] Natella D (2023). A co-estimation framework for state of charge and parameters of Lithium-Ion battery with robustness to aging and usage conditions. IEEE Trans. Ind. Electron..

[32] CTS—Cell Test System Series |—alvatek.co.uk. https://www.alvatek.co.uk/energy/battery-testing/basytec/cts_series/. Accessed 21-11-2023

[33] Birkl CR (2015). A parametric open circuit voltage model for lithium ion batteries. J. Electrochem. Soc..

[34] What is WLTP and how does it work? urlhttps://https://www.wltpfacts.eu/what-is-wltp-how-will-it-work/. Accessed 27-11-2023.

[35] Auger, D. Driving Cycle (Simulink Block). https://uk.mathworks.com/matlabcentral/fileexchange/46777-driving-cycle-simulink-block. Accessed 27-11-2023.

[36] Hales, A. *et al.* Isothermal temperature control for battery testing and battery model parameterization. *SAE Int. J. Electrified Veh.* 10 (2021).

[37] Birkl CR (2015). A parametric open circuit voltage model for lithium ion batteries. J. Electrochem. Soc..

[38] Nikolian A (2018). Complete cell-level lithium-ion electrical ECM model for different chemistries (NMC, LFP, LTO) and temperatures (-5^∘^C to 45^∘^C). Optimized modelling techniques. Int. J. Electr. Power Energy Syst..

[39] Zhao Y  (2018). Modeling the effects of thermal gradients induced by tab and surface cooling on lithium ion cell performance. J. Electrochem. Soc..

[40] Samieian, M. A. *et al.* A novel experimental technique for use in fast parameterisation of equivalent circuit models for Lithium-Ion batteries. *Batteries* **8**(9) (2022).

[41] MATLAB Optimization Toolbox (2023). https://uk.mathworks.com/products/sl-design-optimization.html

[42] Jackey, R. *et al.* Battery model parameter estimation using a layered technique: An example using a lithium iron phosphate cell (2013).

[43] Jackey, R. *et al.* Parameterization of a battery simulation model using numerical optimization methods (2009).

[44] Grandjean TRB (2017). Structural identifiability of equivalent circuit models for Li-ion batteries. Energies.

[45] Hossain, M *et al.* A parameter extraction method for the Thevenin equivalent circuit model of Li-ion batteries. In *2019 IEEE Industry Applications Society Annual Meeting* **2019**, 1–7 (IEEE, 2019).

[46] Ribeiro MI (2004). Kalman and extended Kalman filters: Concept, derivation and properties. Inst. Syst. Robot..

[47] Terejanu, G. A. *et al.* Extended Kalman filter tutorial. In: University at Buffalo, 27 (2008).

[48] Humpherys J (2012). A fresh look at the Kalman filter. SIAM Rev..

[49] Sepasi S (2014). Improved extended Kalman filter for state of charge estimation of battery pack. J. Power Sources.

[50] Chen Z (2012). State of charge estimation of lithium-ion batteries in electric drive vehicles using extended Kalman filtering. IEEE Trans. Veh. Technol..

[51] Jiang, C. *et al.* Extended Kalman Filter based battery state of charge (SOC) estimation for electric vehicles. In *2013 IEEE Transportation Electrification Conference and Expo (ITEC)* **2013**, 1–5 (IEEE, 2013).

